# Parallel PWMs Based Fully Digital Transmitter with Wide Carrier Frequency Range

**DOI:** 10.1155/2013/373429

**Published:** 2013-09-18

**Authors:** Bo Zhou, Kun Zhang, Wenbiao Zhou, Yanjun Zhang, Dake Liu

**Affiliations:** School of Information and Electronics, Beijing Institute of Technology, Beijing 100081, China

## Abstract

The carrier-frequency (CF) and intermediate-frequency (IF) pulse-width modulators (PWMs) based on delay lines are proposed, where baseband signals are conveyed by both positions and pulse widths or densities of the carrier clock. By combining IF-PWM and precorrected CF-PWM, a fully digital transmitter with unit-delay autocalibration is implemented in 180 nm CMOS for high reconfiguration. The proposed architecture achieves wide CF range of 2 M–1 GHz, high power efficiency of 70%, and low error vector magnitude (EVM) of 3%, with spectrum purity of 20 dB optimized in comparison to the existing designs.

## 1. Introduction

Wireless communication is becoming more and more important and ubiquitous in modern society. To support numbers of communication standards in small and same handheld devices, there are growing demands for flexible transmitters and receivers supporting multimode communications with high efficiency. Recently, lots of researches have been conducted in RF reconfigurable transceivers using novel hardware implementation. This paper focuses on fully digital wireless transmitters.

The existing design [1, 2], employing all-digital phase-locked loop (ADPLL) and delta-sigma modulator, introduces large fractional spurs and requires strict energy match between power branches. The existing transmitters with quadrature [3, 4] or delay-line [5, 6] based intermediate-frequency (IF) pulse-width modulation (PWM), are not apt for low carrier-frequency (CF) applications, under which the band-pass filter (BPF) fails to suppress IF component or harmonics closer to the carrier. The existing radio-frequency (RF) PWM [7] or direct digital frequency synthesizer (DDFS) [8] based transmitters, aiming for low CF conditions, are not considered as fully digital designs, since analogue feedback or mixed-signal configurations are used. The existing architecture [9] employing outphasing amplification technique is not widely used in commerce due to strict matching requirements between dual paths and distortion and efficiency degradation caused by RF power combiner. 

In this paper, delay-line based CF-PWM with precorrection logic is proposed, where the only carrier clock is employed to ensure spectrum purity under low CF conditions. By combining IF-PWM for high CF and precorrected CF-PWM for low CF, a fully digital transmitter is presented with wide CF range and high efficiency. A unit-delay autocalibration loop for delay lines is also proposed with reconfigurable carrier frequency *f*_*C*_.

## 2. Architecture

**Figure 1:**
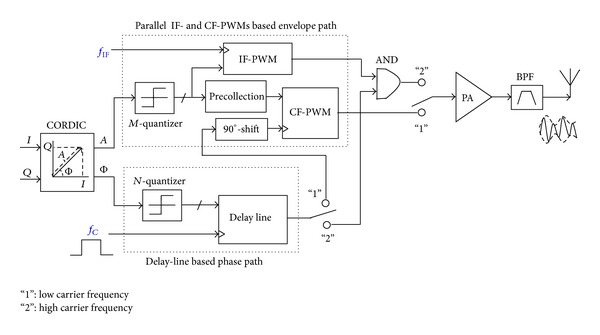
Proposed fully digital transmitter with parallel IF- and CF-PWMs.

Figure 1 shows the proposed fully digital transmitter with parallel IF- and CF-PWMs. The coordinate rotation digital computer (CORDIC) algorithm accomplishes the conversion from *I*/*Q* to polar coordinate *A*/Φ [10]. The wide CF range of 2 M–1 GHz is divided into low *f*_*C*_ band of 2 M–100 MHz and high *f*_*C*_ band of 80 M–1 GHz. About 20 MHz frequency overlap area is chosen to avoid the switching jitter between high and low *f*_*C*_ bands. Under low CF band, the transmitter working in mode “1” and CF-PWM enabling, multibit phase component Φ is mapped to 1/4-period position of the carrier clock, and multibit envelope component *A* is converted to precorrected pulse width of the carrier clock. For high CF case, mode “2” and IF-PWM enabling, phase Φ is conveyed by rising-edge position of the carrier clock, and envelope *A* is represented by pulse width of an IF clock and subsequently converted to pulse density of the carrier clock. The position- and pulse-modulated carrier clock, amplified by a switched-mode class-D power amplifier (PA), is reshaped to the phase- and envelope-modulated sinusoidal carrier by an BPF with CF or IF harmonics suppressed.

The output sinusoidal amplitude *y* and input duty cycle *d* of the BPF conform to (1), according to Fourier series expansion of periodic square wave [7]. For IF-PWM mode, it is the pulse densities rather than pulse widths of the carrier clock that represent baseband envelopes; the BPF handling standard square waves with a fixed duty cycle of 0.5 does not encounter any nonlinear distortion. However, for CF-PWM one, the pulse widths or duty cycles on behalf of baseband envelopes are variable. That is, the BPF not only introduces envelope distortions, but also degrades original rising-edge phases hidden in the modulated carrier due to zero-crossing point deviations. Therefore, to ensure the modulation linearity of CF-PWM mode, an envelope precorrection module with an inverse function of (1) is added to compensate for the envelope offset, and 1/4-period (90°-shifted point) positions rather than rising-edge (zero-crossing point) positions of the carrier clock are modulated to convey phase components without distortion as follows:

(1) $$\begin{array}{c} y=\{\begin{array}{cc} \frac{2}{\pi }, & \text{IF-PWM }\left(d=0.5\right),\\ \frac{2}{\pi }\sin\left(\pi \times d\right), & \text{CF-PWM.} \end{array} \end{array}$$

For CF-PWM mode, only CF clock is involved in signal modulations, whereas IF clock or aliasing is avoided and carrier harmonics are effectively eliminated by BPF. Under IF-PWM condition with less limitation or enough margin between *f*_*C*_ and signal bandwidth, although IF clock *f*_IF_ is introduced, the BPF could still suppress IF interferences by setting reasonable relations between *f*_*C*_, *f*_IF_, and BPF bandwidth *B*_RF_, following (2)

(2) fCBRF≤50,  fIFBRF≥2.5,k+1>fCfIF≠k+0.5>k, k=10~19.

With 6th order BPF, IF spurs lower than −80 dB are ensured when *f*_IF_ is more than 5 times farther than the BPF corner and when the tenth-order or higher IF harmonics with less power locate around the carrier band. With normalized envelope values higher than 0.1, there is at least one whole carrier clock transmitted to convey baseband components during each IF period. In addition, the ratio *f*_*C*_/*f*_IF_ should not be a multiple of 0.5 to avoid IF harmonics located at 2*f*_*C*_ down-converts with the carrier at  *f*_*C*_ into the signal band. Here, *f*_IF_ = *f*_*C*_/15.4.

## 3. Implementation

### 3.1. IF-PWM Mode

**Figure 2:**
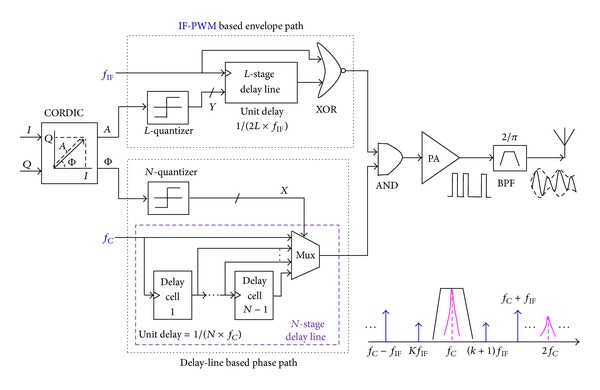
Delay-line based IF-PWM mode for proposed fully digital transmitter.

Figure 2 shows delay-line based IF-PWM mode for the proposed transmitter. Multibit phase components are mapped to the rising-edge positions of the carrier clock with a fixed duty cycle of 0.5 by employing an *N*-level quantizer and an *N*-stage delay line where the continuous phases of 0–360° are equidistantly quantized to *N* discrete values. Multibit envelope components are converted to the pulse widths of the IF clock, which are subsequently converted by AND operation to the pulse densities of the carrier clock with the fixed pulse width inversely proportional to *f*_*C*_. Under enough margins and perfect values between *f*_*C*_, *f*_IF_, and *B*_RF_, the BPF effectively suppresses the IF spurs or harmonics. Modulation linearity strongly depends on the quantization resolutions or levels.

**Figure 3:**
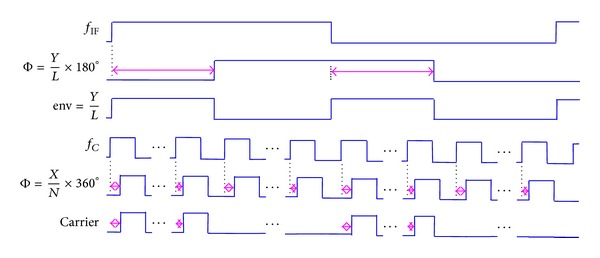
Function diagram of IF-PWM architecture.

The function diagram of the IF-PWM mode is shown in Figure 3. For a certain carrier frequency *f*_*C*_, the *N*-stage delay line with a unit delay of 1/(*N* × *f*_*C*_) converts the quantized phase *X* to the rising-edge lag Φ of the carrier clock. The *L*-stage delay line with a unit delay of 1/(2*L* × *f*_IF_) maps the quantized envelope *Y* into the rising-edge lag Φ of the IF clock. The pulse-width env generated by Φ shapes the pulse density of the carrier clock to represent the envelope component, and the rising-edge lag Φ of the carrier clock reflects the phase component, respectively.

Assuming baseband envelope *A*(*t*), phase Φ(*t*), and envelope normalized value *A*_std_, the modulated carrier clock *c*(*t*) is shown in (3), where square() is the function of periodic square wave and *ω*_*C*_ is the carrier angular frequency. One has

(3) $$\begin{array}{c} c\left(t\right)=\frac{A\left(t\right)}{A_{\text{std}}}\times \text{square}\left[\omega_{c}t+\Phi \left(t\right)\right]. \end{array}$$

After filtered smoothly by the sequent BPF, the modulated sinusoidal carrier *y*(*t*) is shown in (4). Continuous baseband envelope *A* and phase Φ are transmitted:

(4) y(t)=A(t)Astd×2π×sin[ωct+Φ(t)]=2π×Astd[A(t)×sin(ωct+Φ(t))].

### 3.2. Proposed CF-PWM Mode

**Figure 4:**
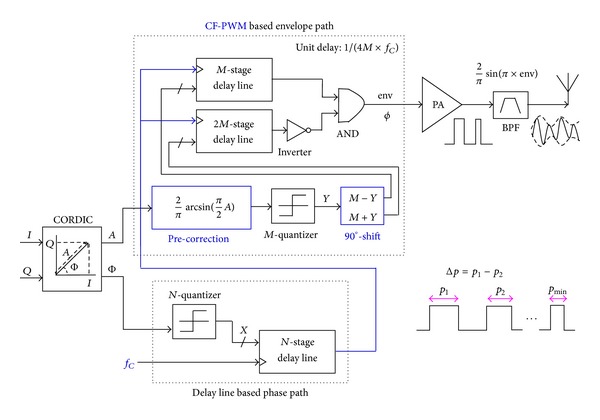
Proposed delay-line based CF-PWM mode for fully digital transmitter.

Figure 4 shows the proposed delay-line based CF-PWM mode for fully digital transmitter. Multibit phase components are mapped to the rising-edge positions of the carrier clock with a fixed duty cycle of 0.5 by employing an *N*-level quantizer and an *N*-stage delay line. Multibit envelope components are converted to the pulse widths of the position-modulated carrier clock through the delay differences between the *M*- and 2*M*-stage delay lines and subsequent logic operations. Unlike IF-PWM mode, it is pulse widths rather than densities of the carrier clock that represent baseband envelopes. For ensuring the modulation linearity, both envelope precorrection module with antitrigonometric function and 90° phase-shifted operation are added to compensate for the distortions caused by the BPF with variable pulse-width inputs, as discussed above.

**Figure 5:**
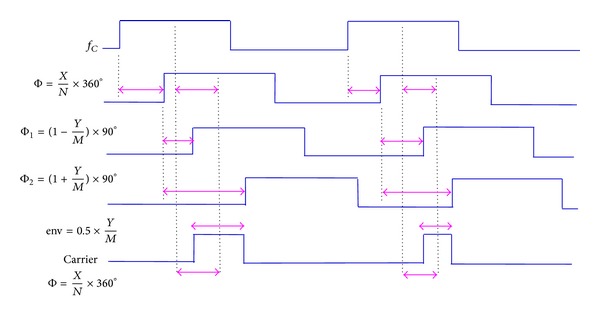
Function diagram of proposed CF-PWM architecture.

The function diagram of the proposed CF-PWM architecture is shown in Figure 5. For a certain carrier clock, the *N*-stage delay line with a unit delay of 1/(*N* × *f*_*C*_) converts the quantized phase *X* to the rising-edge lag of the carrier clock. The *M*- and 2*M*-stage delay lines with a unit delay of 1/(4*M* × *f*_*C*_) subsequently map the precorrected quantization envelope *Y* into two different rising-edge lags Φ_1_ and Φ_2_ on the symmetry of 90° phase-shifted points. The pulse-width env generated by Φ_1-2_ represents the precorrected envelope component, and the lag Φ of the 90° phase-shifted points reflects the phase component, respectively. That is, both pulse-widths and 1/4-period positions of the carrier clock convey baseband signals. Similarly, quantization resolutions or levels determine linearity. The modulated carrier clock *c*(*t*) is shown in the following equation:

(5) c(t)=0.5×YM×square[ωct−90°+Φ(t)]=0.5×[2πarcsin⁡(π2×A(t)Astd)] ×square[ωct−90°+Φ(t)].

The modulated sinusoidal carrier *y*(*t*) is shown in (6). Continuous baseband envelope *A* and phase Φ are transmitted. Consider

(6) y(t)=2π×sin[π×(0.5×[2πarcsin(π2×A(t)Astd)])] ×sin(ωct−90°+Φ(t))=1Astd[A(t)×sin(ωct−90°+Φ(t))].

The minimum pulse width *p*_min⁡_ of the modulated carrier clock, shown in (7), is determined by the maximum CF *f*_*C*,max⁡_ and the minimum normalized envelope *A*_std,min_. The pulse width resolution Δ*p*, shown in (8), depends on *f*_*C*_ and envelope quantization level *M* and needs to be recognized by the sequent PA. Clearly, the ability of the PA conducting narrow pulses is a critical parameter for the proposed implementation. With finite signal rise and fall time, ultra narrow pulses going through the class-D PA are prone to be trapezoidal or triangular rather than being square, considering the limited working frequency or conducting capability of the present PA. That is why the proposed CF-PWM architecture only aims for low CF range of 2 M–100 MHz. Consider the following equations:

(7) $$\begin{array}{c} p_{\min}=\frac{0.5}{f_{C,\max}}A_{\text{std,min}}, \end{array}$$

(8) $$\begin{array}{c} \Delta p=\frac{0.5}{M\times f_{C}}. \end{array}$$

### 3.3. Unit-Delay Autocalibration

**Figure 6:**
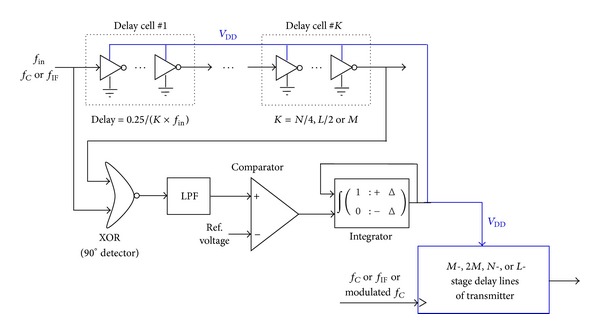
Proposed unit-delay autocalibration loop with reconfigurable *f*_*C*_.

To ensure modulation performances, the unit delay of delay lines needs to be accurate. To meet wide *f*_*C*_ range, the unit delay also needs to be reconfigurable. In addition to D flip-flops apt for large unit delay, in most cases the unit delay is small and is implemented by inverters without or with resistive interpolation [5, 11]. However, inverters require accurate delay calibration for high robustness over process and temperature variations.  Figure 6 shows the proposed unit-delay calibration loop with wide and reconfigurable*f*_*C*_. 

The phase difference between the periodic clock input and 90°-shifted clock corresponding to *K* unit delays is measured and converted to an error voltage by an XOR gate followed by a low-pass filter (LPF) and a sequent comparator. The error voltage sent to an integrator with driver buffer is accumulated and fed to the power supply *V*_DD_ of delay cells, which inversely modifies the unit delay. Under the negative feedback operation with closed-loop *V*_DD_ calibration, for a certain clock *f*_in_ (*f*_*C*_ or *f*_IF_), the unit delays of open-loop delay lines are accurately achieved by strict match designs and conform to (9), respectively, for different delay lines of the phase path and IF- and CF-PWMs. By setting matched inverter number in each delay cell and perfect *V*_DD_ range of delay cells, reconfigurable  *f*_*C*_ and corresponding unit delays are accomplished as follows:

(9) τ=0.25K×fin={1N×fC,Phase-Path (K=N4,fin=fC), 12L×fIF,IF-PWM (K=L2,fin=fIF), 14M×fC,CF-PWM (K=M,fin=fC).

## 4. Experimental Results

The proposed fully digital transmitter is designed in 180 nm CMOS with external class-D PA and BPF. Excluding the PA, the proposed implementation only dissipates ultra low power of 100 *μ*W from 1.8 V power supply. The transmitter achieves a power efficiency of 70%, according to circuit-level simulations. The quantization levels are 256 and 128 for the phase and envelope paths, respectively. 

The supply voltage of delay cells can be changed from 1.4 to 2.2 V, which corresponds to the minimum unit delays of 7.8–3.9 ps for the phase path, 120.3–60.1 ps for the IF-PWM, and 39.0–19.5 ps for the CF-PWM, according to circuit-level simulations of three groups of different delay lines in the nominal case. As a result, the CF ranges of 50–100 MHz for the CF-PWM mode and 0.5–1 GHz for the IF-PWM one are gotten. By adding matched inverters in each group of delay cells, the lower CF range is also covered and the wide CF range of 2 M–1 GHz is achieved. For a certain baseband signal with normalized envelope range of 0.15–1.0, with *f*_*C*_ less than 100 MHz, the *p*_min⁡_ and Δ*p* of the CF-PWM mode are 0.75 ns and 0.04 ns, respectively, which could be recognized by the present PA.

**Figure 7:**
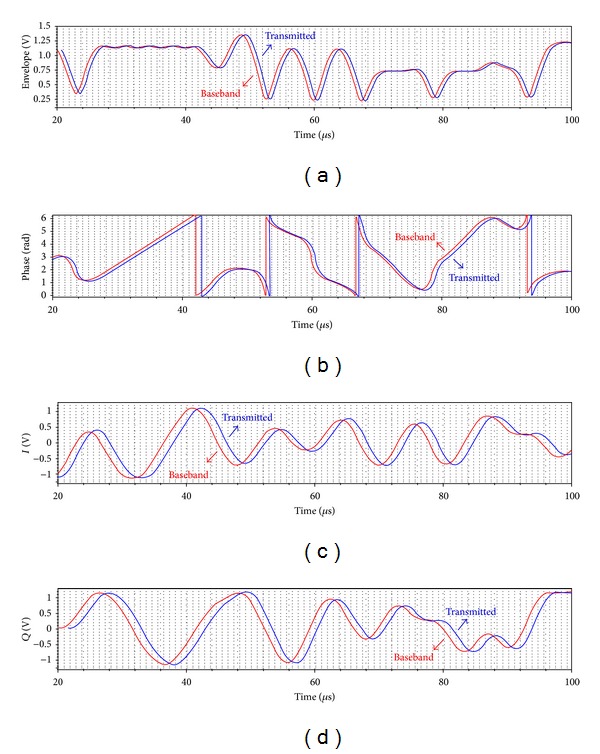
Simulated transmitter components conveyed by proposed architecture with comparison to baseband ones.

Figure 7 shows the simulated transmitter components conveyed by the proposed architecture with comparison to the baseband ones for 8PSK signal with an envelope range of 0.22~1.45 V. The proposed transmitter conveys the baseband components very well with the simulated error vector magnitude (EVM) of 3%. Little distortions result from limited quantization resolutions and slight PA push-pull asymmetry. An ideal receiver reconstructing the transmitted components is implemented in Matlab.

**Figure 8:**
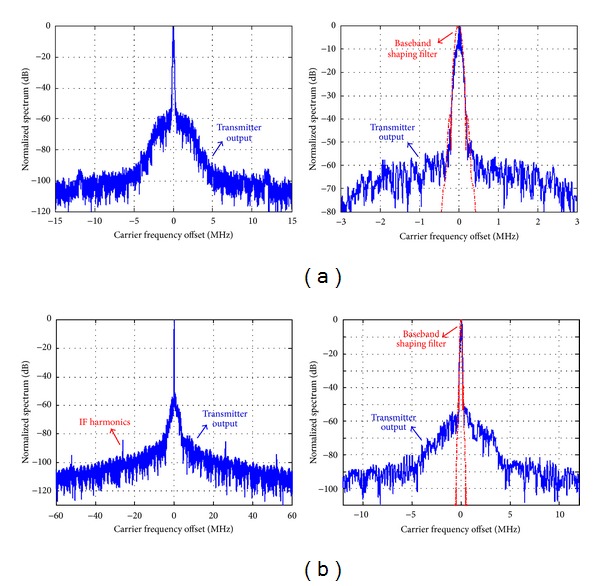
Simulated transmitter normalized far and near spectrums: (a) CF-PWM centered at 50 MHz; and (b) IF-PWM centered at 200 MHz.

Figure 8 shows the simulated transmitter normalized far and near spectrums centered at 50 MHz for CF-PWM mode and at 200 MHz for IF-PWM one. The roll-off feature of spectrum side lobes is determined by the baseband shaping filter. The noise floor less than 60 dB is observed below the carrier, and 20 dB spectrum optimization is achieved when compared to the existing designs [4, 5] with the noise floor at 40 dB below the carrier. For CF-PWM mode, no any other frequency components including spurs and harmonics exist, except for the carrier. For IF-PWM mode, IF spurs located at even multiples of *f*_IF_ from *f*_*C*_ are observed and less than −80 dB. Hence, the proposed transmitter achieves high spectrum purity.

## 5. Conclusions

By combining delay-line based IF-PWM for high *f*_*C*_ band and proposed CF-PWM with precorrection for low *f*_*C*_ band, a fully digital transmitter is implemented in 180 nm CMOS, with wide CF range of 2 M–1 GHz and high power efficiency of 70%. A unit-delay autocalibration circuit with closed-loop detection and open-loop adjustment is presented to support *f*_*C*_ reconfiguration. The experimental results show that the proposed architecture transmits baseband components well with high spectrum purity and low EVM of 3%. Compared to the existing designs, 20 dB spectrum optimization is achieved. Chip prototype will be considered as a future work to further verify the proposed transmitter architecture.
